# The effect of internalized stigmatization on care burden in adolescent psychiatric patients and their parents

**DOI:** 10.3389/fpsyt.2023.1192087

**Published:** 2023-08-16

**Authors:** Funda Gümüş, Havva Kaçan

**Affiliations:** ^1^Department of Nursing, Atatürk Faculty of Health Sciences, Dicle University, Diyarbakır, Türkiye; ^2^Department of Nursing, Faculty of Health Sciences, Kastamonu University, Kastamonu, Türkiye

**Keywords:** internalized stigmatization, care burden, adolescent, parents, nursing, psychiatric nursing

## Abstract

**Introduction:**

The purpose of this study was to determine the effect of internalized stigma perceived by adolescents with mental disorders and their families, on the burden of care on their families.

**Methods:**

The study was conducted in a descriptive and correlational desing with adolescents and their parents who applied to the child and adolescent psychiatry outpatient clinic between March 1 and June 1, 2022. A total 101 adolescents and 101 parents who met the sampling criteria and agreed to participate in the study. Only 1 parent per adolescent was included in the study.

**Results:**

It was found that the mean age of the adolescents was 15.05  ±  (1.80), 57.4% were female and 56.4% were secondary school graduates, and 26.7% of the adolescents, all of whom were unemployed, were diagnosed with a mood disorder, 50.5% had received outpatient treatment previously, and 22.8% had chronic diseases. It was found that the mean age of the parents who participated in the study was 45.53  ±  (6.48), 77.1% were female and 28.7% were secondary school graduates. Also, 22.8% of the parents had a chronic disease, 28.7% had a chronic disease in their family and 32.7% had another family member in need of care, and 17.0% of the parents had received training on mental disorders previously. It was found that the parents’ total means ZBI score was 42.74  ±  (11.92). When the ZBI total scores were examined in terms of sociodemographic variables, no significant differences were found between the groups according to the variables. There was only a weak, positive and significant relationship between the age of the parents and in the present study, it was found that adolescents with mental disorders and their families experienced internalized stigmatization and this stigmatization increased the care burden on parents.

**Discussion:**

Mental health and psychiatric nurses can facilitate this population’s access to treatment by planning and implementing psychosocial interventions to reduce the internalized stigma of children and adolescents and their families.

## Introduction

Adolescence is among the most critical periods of life. In this period when the individual struggles with gaining an identity, mental disorders are common, burdening individuals more than is known and causing disability (1). Moreover, it is reported that 3/4 of mental disorders start previouslyat the age of 24. This brings a large burden on adolescent mental health, health, families, communities, and economies throughout life (2). In previous studies, 22% of adolescents in the USA (3), approximately 20% in Canada experience mental disorders (4), and one in six people in Ireland (5) has serious mental disorders, which is gradually increasing (6). A study conducted in Australia reported thatapproximately half (46.5%) of adolescents with mental problems and their families needed help (7, 8), and adolescents with mental health problems are stigmatized at a rate of 50%–75% (9, 10).

Internalized Stigmatization is the adoption of the stigmatizationopinions of society by individuals. Self-Stigmatization of an individual can be explained as feelings, thoughts, beliefs, and fears, as well as the belief that the individual is dangerous to others or incapable of managing his/her own life (11). It is argued that especially adolescents have a high risk of internalizing negative perceptions of others (12). A person’s facingStigmatization at an early age and internalizing this stigmatization can affect the identity development of the person negatively (13).

Stigmatization in mental treatment is the biggest obstacle to recovery (11), and starting and maintaining treatment (14–16) is an important issue that must be eliminated during the treatment process. Because Stigmatization is an obstacle to help-seeking behavior (8). Untreated mental disorders in adolescence can cause social, behavioral, and academic problems, worsening of symptoms or impairments, comorbidities, suicidal behaviors, and chronic disease onset in adults (3). In many previous studies, it was found that the self-esteem of those who experience internalized Stigmatization decreases, mental well-being is affected, they feel ashamed of their disease, they feel inadequate, they experience feelings of loneliness, anger, and helplessness, and their quality of life deteriorates, their social difficulties increase, and their compliance with treatment deteriorates (16–18). As well as these factors, internalized Stigmatization affects psychological and subjective well-being negatively (19). Also, stigmatizedpeople devalue themselves for reasons such as avoiding social environments, face fear of rejection, fall into despair and lose their self-confidence (20). Because of internalized stigmatization, individuals do not consider themselves worthy to take action in many areas of life such as finding a job and making close friends. This response can cause increased depressive symptoms, adverse health outcomes, and a decrease in recovery rates (16). In brief, stigmatization attitudes toward individuals who are diagnosed with mental disease can increase the severity of psychiatric symptoms, delay in receiving medical support, and decrease compliance with treatment. Identifying the groups that are most associated with Stigmatization can guide actions to reduce this bias and improve patients’ prognosis (21). For this reason, the prevalence of mental disorders in adolescents is an important factor for future health and well-being (22).

Adolescents live with their families and the disease is usually managed by families in many cultures (23). The treatment of mental disorders in adolescents begins either when families find outabout the situation or when the adolescent asks for help by sharing it with the family. However, it is not easy for adolescents to accept this and be involved in the treatment process. Because adolescence is the transition period from childhood to adulthood and it contains many crises, especially identity formation. Adolescents are afraid of Stigmatizationin this period when peer relationships are important and being stigmatized is difficult for adolescents to cope with (16, 24).

Internalized stigmatization not only affects people who have mental problems but also affects families and close circles of people with mental problems. Previous studies conducted with adults contain results consistent with these considerations. In a study on internalized stigmatization, it was reported that adults perceived children with psychological problems as dangerous and incompetent (25). However, parents of children with mental diseases are also exposed to stigmatization, which is both trauma and a factor that increases the care burden for families (26). Becausestigmatization also continues with self-blame for parents. As a result of parental blame, parents may feel that their parenting efforts are inadequate and may have the idea that they are personally responsible for their child’s problems. The experience of being judged by others can contribute to feelings of self-blame and distress for parents (27) occurring when a parent becomes aware of others’ judgments and blame reactions towards them causing self-doubt and social avoidance, believing that they are inadequate parents and responsible for their child’s problems (28). Parents of mentally ill people may blame themselves for having such a child. They might also think that they are not good parents to their children or are inherited from the genes that caused the disease and that this is something to be ashamed of. For this reason, they hide the mental disorder from their surroundings (27, 29, 30). In a qualitative study that was conducted by Dikeç et al. (31) to determine the Stigmatization experiences of the parents of adolescents who were hospitalized for mental disorders, it was found that all parents hid their children’s disease during starting a new job or meeting a new person (31). Individuals and families who have mental disorders experience social isolation because parents avoid social relations because of feelings of guilt or shame, and society maintains a social distance between them and their families (32). Also, individuals who have mental disorders or their families experience stigmatization anxiety even if they are not concretely exposed to stigmatization or discrimination. This perception or worry experienced by family members can increase the care burden for families by causing anxiety and depressive symptoms (33, 34). When parents direct these negative feelings to their children who have mental disorders, this can be even more devastating for individuals with mental disorders than being ostracized or stigmatized by society (30).

It was considered that the internalization of Stigmatization by the people around children and adolescents who have mental disorders may cause internalization of the stigmatization by children and adolescents. When studies that were conducted in Turkey were reviewed, Oban and Küçük (35) reported that adolescents had negative attitudes and prejudices toward mental diseases and that adolescents wanted to maintain social distance from people with psychological problems (35). In a study that investigated internalized stigmatization tendencies of parents and adolescents, it was found that internalized stigmatization scores were similar. Parents who tried to cope with mental problems in adolescence may experience difficulties in family processes. Parents who have an adolescent with a mental problem may think that they are not good parents to their children, and may hide the mental disorder from their surroundingsfor the fear of Stigmatization of their children and themselves, with the thought that their children’s disease is genetically transmitted. For this reason, they hesitate to apply to healthcare institutions, and when the treatment is started, they stop the treatment and cannot maintain their well-being. Individuals who have mental problems and cannot receive adequate treatment and support may harm both their parents and themselves. As a result, it is stated that the internalized stigmatization perception of children-adolescents and their parents affects treatment compliance negatively as a barrier to starting or maintaining treatment (36). Also, individuals who have mental disorders or their families are worried about Stigmatization even if they are not exposed to stigmatization or discrimination. This perception or anxiety experienced by family members may increase the care burden forfamilies by causing anxiety and depressive symptoms (37). Even if there is no direct stigmatization, they may still be concerned about Stigmatization, which may affect the care burden of parents (38).

Care burden can be defined as the burden experienced by family members who have a chronic illness, disability, child or care for an elderly family member. The concept of “caregiver” is used in the literature for the person who meets the basic needs by living with the patient in need of care or devoting a part of his time to him (39). The caregiver burden is affected by all restrictions in the healthcare system, social Stigmatization, social life, symptoms of a disorder or disease of the caregiver, economic strains, and personal healthcare problems of caregivers. Parents may hide individuals who have mental problems during adolescence because they might think that they will be stigmatizedbecause of their school life or career acquisition. They perceive this as guilt or shame and try to restrict it from social life (17). In our country and international literature, no study was found comparing the levels of mental disease internalized stigmatization of adolescents and their parents and reveals the effect of internalized stigmatization on the care burden. By determining the internalized stigmatization levels of both groups, data can be provided for mental healthcare professionals to ensure compliance with the treatment of patients and their relatives, and suggestions will be made to provide psychosocial support by determining the relationship between internalized stigmatization of parents and the care burden.

The purpose of the study was to determine the effects of internalized Stigmatization levels of adolescents and their parents applying to the child and adolescent psychiatry outpatient clinic on the care burden.

## Materials and methods

The present study is a descriptive-correlative study examining the effects of internalized. Stigmatization levels of adolescents and their parents who applied to the child and adolescent psychiatry outpatient clinic on the care burden.

### Participants and recruitment

The population of the study consisted of adolescents, who were aged 12–18 years, used psychotropic drugs for at least 3 months and were followed up with a diagnosis of any mental disorder according to DSM-V in the child and adolescent psychiatry outpatient clinic between March 01 and June 01, 2022, and their parents. Only one parent of each adolescent was included in the study. Adolescents that presented with the diagnosis of intellectual disability, pervasive developmental disorder, learning disability, speech and adjustment disorder, attention deficit hyperactivity disorder, and their parents were not included. A total of 101 adolescents, who applied to the outpatient clinics with their parents at the time of the study, who met the sample selection criteria, and who agreed to participate in the study, and 101 parents, who were aged between 18–65 years, who did not have any mental disorder and agreed to participate in the study, were included in the study. The data collection was performed by the researchers in an empty polyclinic room using the face-to-face interview technique. Each interview lasted approximately 10–20 min. Adolescents and parents who wanted to fill the forms themselves were allowed for this, and necessary explanations were made previously the procedure.

### Measures

Two information forms were prepared in the study to collect the data on sociodemographic and mental disorders for adolescents and their parents. There were a total of 7 questions in the Information Form-Adolescent prepared for adolescents, and 9 questions in total in the Information Form-Parent (40). Also, the internalized Stigmatization levels of adolescents were found by the *Internalized Stigma of Mental illnessScale–Adolescent Form(ISMI-AF)*, and the parents by the *Parents’ Internalized Stigma of Mental Illness Scale (PISMI).* The *Zarit Burden Interview (ZBI)* was used to determine the care burden.

#### Parents’ internalized stigma of mental illness scale

The Parent’s Internalized Stigmatization in Mental Disease Scale that was developed by Dikeç et al. (41) was used in the study. The scale consisted of 29 items in a 4-point Likert style, based on self-report. The scale includedfive subscales “alienation (items: 1,5,8,16,17,21)”, “confirmation of stereotypes (items: 26,10,18,19,23,29)”, “perceived discrimination (items: 3, 15,22,25,28)”, “social withdrawal (items: 4,9,11,12,13,20)”, and “resistance to Stigmatization (items: 7,14,24,26,27)”. The items on the scale are answered as “I strongly disagree” (1 point), “I disagree” (2 points), “I agree” (3 points), and “I strongly agree” (4 points). The items (7,14,24,26,27) of the resistance to Stigmatization subscale are calculated inversely. The total ISMI-AF score obtained by adding the five subscales ranges from 29 to 116 and there is no cut-off score for the scale. High scores show that the internalized Stigmatization of the individual is more severe in a negative way (41). In the present study, Cronbach’s Alpha Coefficient for the scale total score was found to be 0.86.

#### Internalized stigma of mental illnessscale–adolescent form

The scale consisted of 29 items in a 4-point Likert type and was based on self-reporting. The scale included5 subscales “alienation (items: 1,5,8,16,17,21)”, “confirmation of stereotypes (items: 26,10,18,19,23,29)”, “perceived discrimination (items: 3, 15,22,25,28)”, “social withdrawal (items: 4,9,11,12,13,20)”, and “resistance to Stigmatization (items: 7,14,24,26,27).” The items on the scale are answered as “I strongly disagree” (1 point), “I disagree” (2 points), “I agree” (3 points), and “I strongly agree” (4 points). The items (7,14,24,26,27) of the resistance to Stigmatization subscale are calculated reversely. The total score is obtained by dividing the sum of the five subscales by the number of items. The total score ranges from 1 to 4 and there is no cutoff score. High scores show that the internalized stigmatization of the individual is more severe in a negative way (42). In this study, Cronbach’s Alpha Coefficient for the total score was calculated as 0.88.

#### Zarit burden interview

The ZBI is a data collection tool that was developed by Zarit, Reever, and Bach-Peterson (43) to evaluate the difficulties faced by caregivers and to uncover this situation. The adaptation studies of the scale into Turkish were conducted by Inci (44). The ZBI consisted of 22 items applied without a time limit. The scale has a 4-point Likert-style rating ranging from 0 to 4 as “never”, “rarely”, “sometimes”, “often”, and “almost always.” A minimum score of 0 and a maximum score of 88 can be obtained from the scale, and a high score means a high level of distress [Zarit and Zarit, 1990 (45)]. The version of the scale adapted to Turkish consists of one dimension. The internal consistency coefficient of the scale was found to be 0.95. In the present study, it was found that the internal consistency coefficient of the scale was 0.87, which shows that the scale is a reliable measurement tool to be used in the statistical analysis process (44). In the present study, Cronbach’s Alpha Coefficient was found to be 0.86.

### Analysis

The study data were analyzed in the SPSS 25.0 program. Mean, standard deviation, minimum, maximum, number, and percentage were used in the analysis of the descriptive data. By taking the mean total score of the scales, it was examined whether the scales showed normal distribution with Kurtosis and Skewness, and it was found that the scale scores showed normal distribution. The independent Student *t*-test used for those with two groups in the independent variables and ANOVA test were used for those with three or more groups in the independent variables to analyze the sociodemographic variables according to the mean ZBI total scores. The Pearson Correlation Test was used to examine the relationship between ZBI, age variables, and scales. The Multiple Linear Regression Analysis was performed and the Backward Method was used to examine the effects of PISMI, ISMI-AF, and the significant correlation of parent age variable on ZBI. The Cronbach’s Alpha Coefficient was calculated in the internal consistency analysis of the scales. Paired student t test was used for pretest and posttest measurements of dependent variables. All findings were evaluated at *p* < 0.05 significance level.

## Results

The sociodemographic characteristics of the adolescents who participated in the study and their parents are given in Table 1. It was found that the mean age of the adolescents was 15.05 ± 1.80, 57.4% were female and 56.4% were secondary school graduates, and 26.7% of the adolescents, all of whom were unemployed, were diagnosed with a mood disorder, 50.5% had received outpatient treatment previously, and 22.8% had chronic diseases. It was found that 89.1% of the adolescents had not received training on mental disorders previously, and 12.9% of them had previously received inpatient treatment in a psychiatry outpatient clinic.

**Table 1:** Socio-demographic characteristics of the participants and comparison according to ZBI.

	Adolescents	Parents
**Characteristics**	**Mean (SD)**	**Mean (SD)**
**Age**	15.05 (1.80)	45.53 (6.48)
***r*/*p***	*r*: −0.74/*p*:0.46	*r*: 0.22*/*p*:0.02
**Gender**	***n* (%)**	***n* (%)**
Female	58 (57.4)	78 (77.1)
Male	43 (42.6)	23 (22.8)
***t*/*p***	*t*: 0.75/*p*:0.43	*t*: 0.83/*p*:0.40
**Education status**		
Illiterate	4 (4)	2 (2)
Literate	5 (5)	16 (15.8)
Primary school	13 (12.9)	24 (23.8)
Middle school	57 (56.4)	29 (28.7)
High school	22 (21.8)	17 (16.8)
University	–	13 (12.9)
***F*/*p***	*F*: 1.75/*p*:0.14	*F*: 1.55/*p*:0.17
**Residence**		
City		82 (81.2)
Village		19 (18.8)
***t*/*p***		*t*: −0.35 /*p*:0.78
**Economic status**		
Low		12 (11.9)
Moderate		67 (66.3)
High		22 (21.8)
***F*/*p***		*F*: 1.26/*p*:0.28
**Employment status**		
Employment	–	33 (32.7)
Unemployment	101 (100)	68 (67.3)
***t*/*p***		*t*: 0.98/*p*:0.32
**Diagnosis of mental illness**		
Substance use disorders	15 (14.9)	
Conduct disorders	10 (9.9)	
Mood disorders	27 (26.7)	
Anxiety disorders	23 (22.8)	
Conduct + mood disorders	13 (12.9)	
Anxiety disorders + conduct disorders	2 (2)	
psychotic disorders	11 (10.9)	
***F*/*p***	*F*: 0.92/*p*:0.48	
**Received outpatient treatment**		
Yes	51 (50.5)	
No	50 (49.5)	
***t*/*p***	*t*: −0.64/*p*:0.51	
**Any chronic diagnoses**		
Yes	23 (22.8)	23 (22.8)
No	78 (77.2)	78 (77.2)
***t*/*p***	*t*: 1.31/*p*:0.19	*t*: 1.01/*p*:0.31
**Family member with chronic disease**		
Yes		29 (28.7)
No		72 (71.3)
***t*/*p***		*t*: 0.17/*p*:0.86
**Member responsible for care**		
Yes		33 (32.7)
No		68 (67.3)
*t*/*p*		*t*: 1.31/*p*:0.19
**Training about mental illness**		
Yes	11 (10.9)	14 (13.9)
No	90 (89.1)	87 (86.1)
***t*/*p***	*t*: 0.15/*p*:0.87	*t*: −1.77/*p*:0.08
**Psychiatric hospitalization**		
Yes	13 (12.9)	
No	88 (87.1)	
*t*/*p*	*t*: 0.15/*p*:0.87	

SD: standard deviation: correlation is significant at the 0.05 level. *r*: pearson correlation test. *F*: ANOVA test. *t*: student *t*-test.

It was found that the mean age of the parents who participated in the study was 45.53 ± 6.48, 77.1% were female and 28.7% were secondary school graduates. It was found that 81.2% of the parents lived in the city, 66.3% of them perceived their economic status as moderate and 32.7% of them worked in a job. Also, 22.8% of the parents had a chronic disease, 28.7% had a chronic disease in their family and 32.7% had another family member in need of care, and 17.0% of the parents had received training on mental disorders previously. It was found that the parents’ total meansZBI score was 42.74 ± 11.92 (min: 9, max: 82). When the ZBI total scores were examined in terms of sociodemographic variables, no significant differences were found between the groups according to the variables. There was only a weak, positive and significant relationship between the age of the parents and ZBI (Table 1).

Table 2 shows the internalized Stigmatization scores of the adolescents and their parents. In this respect, although no significant differences weredetected between the groups in terms of mean ISMI-AFand PISMItotal scores, subscales of alienation, perceived discrimination, and social withdrawal, a significant difference was found between groups in terms of confirmation of stereotypes and resistance to stigmatization (*p* < 0.001).

**Table 2:** Comparison of ISMI-AF and PISMI total score and subscales scores.

	Adolescents	Parents		
Scales	Mean (SD)	Mean (SD)	Test*	*p*
Total score	2.31 (0.49)	2.39 (0.43)	1.52	0.13
Alienation	2.48 (0.73)	2.35 (0.58)	−1.60	0.11
Perceived discrimination	2.21 (0.56)	2.21 (0.54)	0.48	0.63
Stereotype-endorsement	2.1 (0.63)	2.35 (0.63)	**3.18**	**<0.001**
Social withdrawal	2.32 (0.67)	2.27 (0.60)	−0.58	0.55
Stigma resistance	2.41 (0.55)	2.77 (0.49)	**5.54**	**<0.001**

*Student *t*-test.

The correlation between the total mean scores of ISMI-AF, PISMI, and ZBI is given in Table 3. A weak and positive correlation was detected between ISMI-AF and PISMIPISMI and ZBI, and ISMI-AF (as each variable increased, the other variable also increased). In the multiple regression analysis made to determine the explanatory power of the factors that affectedZBI, the model established with ISMI-AF, PISMI and the average age of the parents was statistically significant (*F*:8.42, *p* < 0.001). It was also found that the model with significant explanatory power on ZBI was ISMI-AF and PISMI and these two variables explained 19.4% of ZBI and 20% together with the age of the parents (Table 4).

**Table 3:** Correlation between total scores of scales.

Scales	Adolescents		Parents		ZBI	
	*r*	*p*	*r*	*p*	*r*	*p*
ISMI-AF			0.38*	**<0.001**	0.35*	**<0.001**
PISMI	0.38*	**<0.001**			0.37*	**<0.001**
ZBI	0.35*	**<0.001**	0.37*	**<0.001**		

*r*: pearson corelation. *Corelation is significant at the 0.01 level.

**Table 4:** The effect of ISMI-AF, PISMI and age of parents on ZBI.

Dependent variable	Independent variables	*B*	*ß*	*t*	*p*	*F*	Model (*p*)	*R*^2^
ZBI	Constant	2.95		0.32	0.00	8.42	**<0.001**	0.20
	ISMI-AF	6.23	0.25	2.28	**0.02**			
	PISMI	6.45	0.23	2.63	**0.01**			
	Age of parents	0.21	0.11	1.23	0.22			

## Discussion

In the present study, it was found that the adolescents facedinternalized stigmatization at a rate of 2.31 ± 0.49 and their parents at 2.39 ± 0.43. In the study, when the internalized stigmatization levels of adolescents and parents were examined, it was found that the internalized stigmatization scores were similar, but there was a significant difference between the groups in the subscales of confirmation of stereotypes and resistance to stigmatization. It was reported in the literature that adolescents experienced high internalized stigmatization levels. Kaushik et al. (46) conducted a study with 156 children in South London, it was found that self-Stigmatization was high, especially in Social Values Loss (2.6 ± 0.54), Confidentiality (2.85 ± 0.59), and Self-Stigmatization (2.7 ± 0.70) subscales (46). Similarly, it was reported in a study conducted in Pakistan that adolescents experienced high levels of internalized stigmatization and scored high on Social Value loss (2.27 ± 0.58), Confidentiality (2.70 ± 0.69), and Self Stigmatization (2.13 ± 0.87) subscales (47). In England (2016), it was found that more than half of the adolescents who had mental problems facedstigmatization and discrimination. For this reason, they are unwilling to receive treatment. In a study that was conducted in Singapore (2017), it was found that adolescents facedstigmatization, which is affected by cultural characteristics (48). In a study conducted in Jordan, it was reported that the stigmatization perceived by adolescents was higher than the stigmatization they internalized (49). Chen and Shu (50) also reported in their study conducted with adolescents that adolescents felt different, confused, and stigmatized (50). Kranke et al. (51) reported that 90% of adolescents who used psychiatric drugs experienced internalized stigmatization (51). Moses (52) reported in his study that 25% of adolescents who were receiving treatment for mental disease felt ashamed because of behavioral or emotional problems (52). The internalized stigmatization experienced by adolescents is an obstacle to help-seeking behavior (8) and affected the treatment negatively, and brought extra burdens to the adolescent and the family. Also, these stigmatization experiences affect not only adolescents but also their parents. However, a limited number of studies were found in the literature examining the discrimination and stigmatization of adolescents with mental disorders and their parents. Dikeç et al. (2020) (53) reported that adolescents and parents experienced internalized stigmatization and this stigmatizationwas similar, and only adolescents had higher alienation subscale scores (54). In thepresent study, it was found that parents scored higher on Stereotype-endorsement and stigmatization resistance subscales. It is considered that this may be either because families approve of stereotypes in society or the fact that they are more exposed to social stigmatization or are aware of the stigmatizationthey face. Also, the difference in the resistance to stigmatization subscale is considered to be the fact that families cope with stigmatization better than adolescents with their experiences. This aspect could not be discussed in detail because there is no direct study on this subject in the literature.

Dikec et al. (31) found that all participating parents were stigmatized in many areas and tried to hide their child’s diagnosis (31). Oz et al. (37) conducted a study with mothers of children and adolescents who had Autism Spectrum Disorder (ASD) (*N* = 69) and examined the relationship between mothers’ internalized stigmatization perception, depressive symptom level, anxiety symptoms, and quality of life. The level of internalized stigmatization was found to be moderate. It was also found that most of the mothers had low quality of life scores and also low life satisfaction scores (37). Özaslan and Yıldırım (55) conducted a study with mothers of children who had attention deficit hyperactivity disorder andfound that mothers experienced internalized stigmatization (55). In the study of Uz and Kaya on the stigmatization of children with autism and their families in 2018, mothers said that they were exposed to hurtful glances and words because of their children’s behaviors, some of them laughed while others looked at their children with pity, and there were even those who shouted at their children saying “crazy.” They also stated that they were stigmatized as having autism by their spouse, close friends, and other people because they did not talk to their child, did not take care of him, left him alone, and could not raise him properly. It was observed that some of the mothers who were stigmatized in this way later internalized all these stigmatizations because they considered that these discourses were justified and that “the crime” was caused by themselves and could not realize the situation of their child earlier (56). Kinnear et al. (57) conducted a study with parents of 502 autistic children. Almost all parents reported stigmatization processes. It was alsofound that the children experienced feelings of isolation and exclusion from their friends and families (57). Yüksel and Tanrıverdi (58) conducted a study examining the social problems experienced by families with children with special needs and reported that families avoided social relations because they were excluded by society and felt guilty (58). However, when parents direct these negative feelings to their children who have mental disorders, this can be even more devastating for individuals who have mental disorders than being ostracized or stigmatized by society. In their review, Mukolo et al. (59) emphasized that children’s stigmatization experiences were related to how parents or caregivers perceived children’s emotional or behavioral problems and how they coped (59). It is very important to identify and strengthen the social problems of families. It is considered that strengthening the aspects of social support will increase the courage of families in problem-solving and seeking support, and in this way, their mental health will be affected positively (58). Because in previous studies that were conducted with caregivers, it was reported that the care burden is related to the frequency of depression symptoms and the anxiety experienced by the caregivers can increase their depression (60). It was reported in another study in the literature that there is a relationship between internalized stigmatization, frequency of depressive symptoms, and family burden in primary caregivers of individuals with mental problems (33).

In the present study, it was found that as the internalized stigmatization of the adolescent increased, the internalized stigmatization and care burden of the parents increased and these variables were affected by the age of the parent. No study in the literature directly investigated the relationship between these variables. However, this result will be discussed with the data obtained from previous studies. When the literature was reviewed, it was seen that the age of the caregiver is among the factors affecting the care burden giving. Studies report that the caregiving burden of young parents is higher than that of older parents (61–63). In the thesis study that was conducted by Atar (64), it was reported that variables such as mother’s age, mother’s education level, family education/counseling status, and discrimination/exclusion affect the caregiving burden of the mother increased the care burden of the mother, in other words, the care burden increased as the mother’s age increased (64). In the thesis study conducted by Çandır (65), it was found that maternal age, income status, healthcare issues, the time elapsed after the diagnosis of autism, having a disease other than autism, and autism level of the child increased the care burden in the mothers of children followed up with autism diagnosis. When the mean age groups of mothers were compared with the mean score of caregiving burden, the mean score of caregiving burden of mothers who were under the age of 20 was 38.91, and the mean score of caregiving burden was found to be 50.88 in the 30–39 age group (65). As a result, firstly, the power of a younger mother is higher than that of an older mother. For this reason, being older may cause the mother to become weaker and tired more quickly. Also, it is expected that different responsibilities of mothers appear with the advancement of age and these responsibilities affect the care burden giving. On the other hand, the fact that the young mother has a lot of energy and less experience, sometimes makes the child’s care more emotional and may perceive it as a big burden. The inability to reach studies conductedon this subject brings to mind the idea that such studies are needed in the field and it is recommended to carry out such studies.

## Conclusion

In previous studies, the need for counseling, knowledge, and skills training is expressed more than drug treatment. These services should be expanded in the healthcare system and included in treatment guidelines [Lustig et al., 2022 (8)]. Mental diseases are common, often cause disabilities, and are costly, but also treatable and sometimes preventable diseases that negatively affect the patient and their families. The most important problem with mental diseases is that individuals do not seek help for suchdiseases. The biggest obstacle to the request for help is Stigmatization (8). In the present study, similar to the literature data, it was found that Stigmatization was internalized by adolescents and their families and increased the care burden. In line with this result, the study will guide mental health and psychiatric nurses in making these interventions permanent by first identifying the difficulties faced by adolescents and their families regarding Stigmatizationand care, developing appropriate psychosocial programs to combat such difficulties, and sharing the results of the relevant interventions with mental healthcare professionals. For this reason, preventive studies should be conducted about internalized stigmatization, and help-seeking behavior should be supported in all children and adolescents with mental disorders.

In the present study, it was found that adolescents who had mental disorders and their families experienced internalized Stigmatization, which increased the care burden on parents. It was reported in the literature that individuals in the 12–25 age group, where mental diseases are most common, and their families cannot access mental healthcare services adequately, especially in developed and wealthy countries, because of reasons such as Stigmatization, and adolescents and their families need such services (2). In light of this information, there is a need for qualitative studies to deal with the Stigmatization and internalized Stigmatization experiences of children and adolescents from family and society, the care burden for families, and experimental studies aimingat reducing internalized stigmatization and the care burden. On the other hand, developing national/state-run school mental healthcare programs can help identify such children who have emotional and behavioral problems and provide appropriate psychosocial interventions at the school level (66).

### Limitations

The limitation of the study was that the results cannot be generalized because the study was conducted in one single center. The second limitation of the study was not included ADHD, adjusment disorders and pervasive developmental disorders. Because it is known that these are the most studied subjects in the literature and that the burden of stigma and care is high.

Strength of the study: however, the fact that internalized stigmatization and care burden were investigated simultaneously in both children and their parents shows the power, scientific value, and originality of this study.

## Data availability statement

The raw data supporting the conclusions of this article presented in the study can be made available by the authors. Further questions can be directed to the corresponding authors.

## Ethics statement

The studies involving humans were approved by the ethics committee's permission to conduct the study was obtained from the non-interventional clinical ethics committee of a state university with the decision number -2022-KAEK-7 dated 23/02/2022 and institutional permission from the hospital where the study was conducted. Adolescents, who had mental disorders, participated in the study, and their parents were informed and their written and verbal informed consents were obtained. The studies were conducted in accordance with the local legislation and institutional requirements. Written informed consent for participation in this study was provided by the participants' legal guardians/next of kin.

## Author contributions

FG conceived, designed, and initiated the study. HK was collected data. FG and HK undertook the statistical analysis. The article was written by FG and HK. All authors contributed to the article and approved the submitted version.

## Conflict of interest

The authors declare that the research was conducted in the absence of any commercial or financial relationships that could be construed as a potential conflict of interest.

## Publisher’s note

All claims expressed in this article are solely those of the authors and do not necessarily represent those of their affiliated organizations, or those of the publisher, the editors and the reviewers. Any product that may be evaluated in this article, or claim that may be made by its manufacturer, is not guaranteed or endorsed by the publisher.
